# Polymorphism in Tmem132d regulates expression and anxiety-related behavior through binding of RNA polymerase II complex

**DOI:** 10.1038/s41398-017-0025-2

**Published:** 2018-01-10

**Authors:** Roshan R. Naik, Sergey V. Sotnikov, Rebekka P. Diepold, Stella Iurato, Patrick O. Markt, Andrea Bultmann, Nadine Brehm, Tobias Mattheus, Beat Lutz, Angelika Erhardt, Elisabeth B. Binder, Ulrike Schmidt, Florian Holsboer, Rainer Landgraf, Ludwig Czibere

**Affiliations:** 1 Max Planck Institute of Psychiatry, 80804 Munich, Germany; 2 Department of Behavioral and Molecular Neurobiology, University of Regensburg, Regensburg, Germany; 3 Department of Normal Physiology, Sechenov First Moscow State Medical University, Moscow, Russia; 4 Institute of Physiological Chemistry, University Medical Center of the Johannes Gutenberg University, Mainz, Germany; 5 Present Address: Lee Kong Chian School of Medicine, Nanyang Technological University, Singapore, 308232 Singapore; 6 Present Address: Labor Becker und, 81671 Munich, Germany

## Abstract

*TMEM132D* is a candidate gene, where risk genotypes have been associated with anxiety severity along with higher mRNA expression in the frontal cortex of panic disorder patients. Concurrently, in a high (HAB) and low (LAB) trait anxiety mouse model, *Tmem132d* was found to show increased expression in the anterior cingulate cortex (aCC) of HAB as compared to LAB mice. To understand the molecular underpinnings underlying the differential expression, we sequenced the gene and found two single-nucleotide polymorphisms (SNPs) in the promoter differing between both lines which could explain the observed mRNA expression profiles using gene reporter assays. In addition, there was no difference in basal DNA methylation in the CpG Island that encompasses the HAB vs. LAB *Tmem132d* promoter region. Furthermore, we found significantly higher binding of RNA polymerase II (POLR2A) to the proximal HAB-specific SNP (rs233264624) than the corresponding LAB locus in an oligonucleotide pull-down assay, suggesting increased transcription. Virus mediated overexpression of *Tmem132d* in the aCC of C57BL/6 J mice could confirm its role in mediating an anxiogenic phenotype. To model gene–environmental interactions, HAB mice exposed to enriched environment (HAB-EE) responded with decreased anxiety levels but, had enhanced *Tmem132d* mRNA expression as compared to standard-housed HAB (HAB-SH) mice. While LAB mice subjected to unpredictable chronic mild stress (LAB-UCMS) exhibited higher anxiety levels and had lower mRNA expression compared to standard-housed LAB (LAB-SH) mice. Chromatin immunoprecipitation revealed significantly higher binding of POLR2A to rs233264624 in HAB-EE, while LAB-UCMS had lower POLR2A binding at this locus, thus explaining the enhanced or attenuated expression of *Tmem132d* compared to their respective SH controls. To further investigate gene–environment interactions, DNA methylation was assessed using Illumina 450 K BeadChip in 74 panic disorder patients. Significant methylation differences were observed in two CpGs (cg26322591 and cg03283235) located in TMEM132D depending on the number of positive life events supporting the results of an influence of positive environmental cues on regulation of *Tmem132d* expression in mice.

## Introduction

Anxiety is a state of emotional anticipation to uncertain aversive cues, which helps the organism to adapt to its environment by promoting vigilance and facilitating avoidance behavior^1^. In its normal form, anxiety is a common part of human emotional experience. However, in its pathological form, excessive and inappropriate feelings of anxiety can severely disrupt routine functions leading to anxiety disorders. State anxiety is an immediate unpleasant emotional response to a threatening stimulus, while trait anxiety is the existence of more stable and long-term individual response patterns to anxious stimuli. Anxiety disorders are among the most prevalent class of mental disorders and an estimated 20% of the population are afflicted with it at some point in their lifetime^2^. Both genetic and environmental factors have been shown to be important in mediating anxiety-related behavior. Frequency analysis of anxiety disorders in monozygotic and dizygotic twins reveals that ~30–40% of the variance in occurrence seen between individuals can be attributed to genetic variation, while the remaining 60–70% can be attributed to differences in environment^3,4^. Thus. there is a cross-talk between environmental influences and genetic susceptibility factors that interact to mediate anxiety-related behavior. The study of these interactions is important because they can induce plastic, but persistent structural and functional changes in the brain that underlie susceptibility to anxiety and can explain the inter-individual variability in perception of anxiety. Environment enrichment (EE), includes a combination of inanimate and social stimulation as part of housing conditions. It enhances sensory, motor and cognitive abilities in laboratory animals in comparison to their counterparts housed under standard conditions^5,6^. Previous studies have shown that EE causes a decrease in anxiety in mice and is accompanied by lower *Crhr1* mRNA expression in the basolateral amygdala (BLA), an important gene and an anatomical site involved in mediating anxiety-like behavior^7,8^. On the other hand, stress is a well-known etiological factor and thus unpredictable chronic mild stress (UCMS) has been utilized to model anxiety- and depression-like behavior^9^ and was found to cause increase in *Crhr1* mRNA expression in the BLA of chronically stressed mice^8^. Functional neuroimaging studies in humans have implicated altered activity changes in the anterior cingulate cortex (aCC) or the hippocampus, both closely connected to the amygdala to modulate fear and pathological anxiety responses^10–12^.

The TMEM132D (KIAA1944, MOLT) is a single-pass transmembrane protein with a molecular weight of ~130 kDa and although the precise function of the protein is unknown, it is suggested to serve as a cell-surface marker for oligodendrocyte differentiation^13^. RNA-seq transcriptome analysis in mouse cerebral cortex tissue indicates that *Tmem132d* is expressed in neurons and oligodendrocytes^14^. *TMEM132D* is more expressed in the cortical regions of the human brain and single-nucleotide polymorphisms (SNPs) (rs11060369, rs7309727) have been associated with panic disorder (PD) and anxiety severity^15–17^. One of these common variants or risk genotype (rs11060369) has been associated with higher *TMEM132D* mRNA in the frontal cortex in postmortem brain tissue^15^. Similarly, *Tmem132d* mRNA is higher expressed in the aCC of high (HAB) compared to low (LAB) anxiety-related behavioral mice^15^. Apart from these common variants, even private coding mutations have been associated with pathological anxiety^18^. A recent study also reported higher anxiety and higher amygdala volume in carriers of the variant (rs11060369) in normal healthy volunteers, suggesting a more generic role of the gene in threat processing^19^. Additionally, a study investigating the quantitative trait loci in mice detected *Tmem132d* as suggestive locus for fear acquisition^20^. The goal of this study was first to dissect the functional and molecular mechanism underlying differential expression of the *Tmem132d* gene, and further to investigate gene–environmental interactions in the context of EE and UCMS in the HAB/LAB mouse model of anxiety-related behavior.

## Materials and Methods

### Animals

The animal studies were conducted in accordance with the guidelines of the European Union (Council of the European Communities Directive 2010/63/EU) and were approved by the Institutional Animal Care and Use Committee and local authorities (Government of Upper Bavaria). The HAB and LAB animals, which spent <15% (HAB) or >60% (LAB) of the total test time on one of the open arms of the EPM were bred for over 45 generations in the animal facility of the Max Planck Institute of Psychiatry (MPIP). Suitable numbers of HAB×LAB crosses were made to receive F1 offspring for behavioral and molecular studies. CD-1 and C57BL/6 J mice were purchased from Charles River (Sulzfeld, Germany). Unlike CD-1 mice, C57BL/6 J is an inbred mouse strain and is the preferred choice for AAV experiment because of its relatively homogenous genetic background and little phenotypic variability. Thus, any difference in phenotype can be traced back to the respective treatment. All animals were housed under optimal conditions, i.e., a temperature of 23 ± 2°C, a relative air humidity of 60 ± 5% and a 12/12-h light–dark cycle with beginning of the light phase at 8 AM and had access to food pellets (Altromin GmbH, Lage, Germany) and tap water ad libitum. Only male mice were utilized for this study to avoid potential difference in behavioral or molecular effects when female mice are in different stages of estrous cycle. All the behavioral experiments were performed between 08:30 and 11:30 A.M.

## Sequencing of *Tmem132d*

Genomic DNA was isolated from mouse tail tips (about 6mm) using the NucleoSpin Tissue kit (Macherey-Nagel, Düren, Germany) as per manufacturer’s instructions. To identify SNPs in Tmem132d, 8,339 bp of the unspliced transcript, 1,132 bp of the promoter and 103 bp of the downstream enhancer region were analyzed. Due to the size of the unspliced transcript (675,077 bp), only exons, fragments of 100–500 bp around exons, furthermore two conserved intronal sequences from intron 3 (717 and 389 bp) and 839 bp from intron 4 were considered for sequencing. The primers (Table 1; Sigma-Aldrich (Taufkirchen, Germany)) for sequencing were designed using NCBI/Primer-BLAST and *Tmem132d* DNA fragments were amplified using *Taq-*polymerase (Fermentas, St. LeonRot, Germany) as previously described^21^. Subsequently, PCR products were cleaned up and were utilized for sequencing using Big Dye Terminator kit v3.1 (Applied Biosystems, Foster City, CA, USA), and sequences were resolved by capillary electrophoresis on a 3730 DNA analyzer (Applied Biosystems). Sequence analysis and comparison were performed using FinchTV Ver. 1.2 (Geospiza, Seattle, WA, USA) and BioEdit Ver. 7.0.2 (Tom Hall, Ibis Biosciences, Carlsbad, CA, USA) software programs.

**Table 1:** Primers used for sequencing the most relevant fragments of the *Tmem132d* promoter and exons

Primer set No.	Orientation	Primer sequence (5’ → 3’)
1	Forward	TGCTGCCAAGCTGTGATAAA
1	Reverse	TGCGGATAAAAATGGACTGG
2	Forward	AGCCCAAAACGGACTCTCTT
2	Reverse	GGACAGTTCAGGAACCCCTA
3	Forward	TCAGGGACAGGAATTTGAGG
3	Reverse	TTTTTAAGCCCCACCCTTCT
4	Forward	CCAGGAAGGTGGGACCTACT
4	Reverse	CTGAGGACTGGCTCGTGAAT
5	Forward	CCAGCCGGAGTCCTCAGA
5	Reverse	CCACCCACATCCACATCTACT
6	Forward	AAAGTGAGGCTGTGGGTAGC
6	Reverse	GTTCTCGTCCCTGTGGTCAT
7	Forward	CCAACCCATTTGGATTCACT
7	Reverse	TCCTTCAAAGGTGGACTGCT
8	Forward	AGGAGGATTCGAGGAAGAGC
8	Reverse	TCTGCAGACAGTTCCACAGC
9	Forward	TAAACCTGATTCCCCGTGAG
9	Reverse	GCCCTGTGTGGGTTCACTAT
10	Forward	CTTCCTCAAGCTCCTTCTGG
10	Reverse	GGCTACCCTTGGGATATGGT
11	Forward	ACAGGTTAATGCCCTGTTGC
11	Reverse	AGCTTTGCACACTGCACTTC
12	Forward	GGACTGGGAAAGATGTGGAC
12	Reverse	GGTCTGGAGAGTGTGGGAAC
13	Forward	GAGACGTGATAGGCCCATGT
13	Reverse	CCTTTTACAGGGGTCGCATA
14	Forward	TTGTGGATGGTGGTCAGTCC
14	Reverse	CCATTTTTGTCCCCATTTTG
15	Forward	TGTTGCACCAGCGTTGTAC
15	Reverse	ATCTGGCGTCGCTATTATTAG
16	Forward	GAAGCACCGTGTGTCTAAGG
16	Reverse	CCTGGAGCTTGCTGGTCTAC
17	Forward	ACATTTGGCTTCCTGTGACC
17	Reverse	CCAGGCATGGGTTATAGCAG
18	Forward	ATTCCCTGGTCTGTGGACTG
18	Reverse	GGCAGAGTCTAGGCACTTGG
19	Forward	AACCACCTGTCATGCCTCTC
19	Reverse	CCCTGTGTCTCCCATGTATC
20	Forward	TTATAACATGGGATGGCTGG
20	Reverse	GGGATAGAAACCCCTGATGG
21	Forward	ACCCAATTCTTTTCCTCGAC
21	Reverse	TCCACTCTCACTGCTGTTGG
22	Forward	TCTCCCTGATGGCTACATCC
22	Reverse	AACAGGTTTTCCTGGCCTTC
23	Forward	TGACAGGAGGTCCAAAAAGC
23	Reverse	AGTCCAGACCCCTGTCAATG
24	Forward	TCACTCTCACGACTGGGTTG
24	Reverse	GTGTCCCCGTTATACGTGCT
25	Forward	TCAGACGATGGGTGTCCTTC
*25*	Reverse	CTGACCCACACATGCTCAAC

### Allele-specific transcription assay (F1 offspring)

To determine whether differences in the genetic sequence between HAB and LAB mice could directly be translated into the previously observed expression differences, crossbred HABxLAB F1 mice were analyzed for the HAB- and LAB-specific alleles’ expression. Therefore. qPCR assays were designed to rather amplify the HAB- or the LAB-specific allele based on the SNP identified in exon 3: rs36596918 (G(1,747)A) or exon 9: rs13478518 (A(3164)G. In addition, CD-1 animals heterozygous for the respective SNP were included in the analysis. One primer of the respective primer pairs were designed to bind to the specific allele using 3’ end matching and a mismatch base in the penultimate 3′ position of the primer. To correct for assay-specififc differences, results were calculated based on predefined mixtures of HAB and LAB cDNA.

Mice were killed under basal conditions after brief isoflurane anesthesia. Then, the brains were collected and snap-frozen in N-methylbutane and stored at −80 °C until further use. Histological staining and atlases^22^ were used to collect samples from the aCC with tissue sample corers having a diameter of 0.8 mm (Fine Science Tools, Heidelberg, Germany) from 7 × 200 µm slices continuously cooled on dry ice. RNA isolation and qPCR were carried out as decribed before^23^ using primers (Table 2) from Sigma-Aldrich (Taufkirchen, Germany), the Quantifast SYBR Green Kit (Qiagen, Hilden, Germany) on a LightCycler 2.0 (Roche Diagnostics, Mannheim, Germany).

**Table 2:** Primers used for the allele-specific expression (ASE) analysis in carriers of both alleles

Primer	Orientation	Primer sequence (5’ → 3’)
ASE1-e3-HAB	Forward	GCTCTTCAATCTGGGATGTCAG
ASE2-e3-LAB	Forward	GCTCTTCAATCTGGGATGTCAA
ASE3-cmn-e3	Reverse	TCGGAGCCAGCAGATTTCTTC
ASE4-e9-HAB	Forward	CCTTGGGGCTGGGCTTCATGTG
ASE5-e9-LAB	Forward	CCTTGGGGCTGGGCTTCATGTA
ASE6-cmn-e9	Reverse	TCGGGGCTCCCCCATTCCTG

### Sequenom assay (CD-1 mice)

SNPs that were determined by sequencing as described before were validated in 16 HAB vs. 15 LAB mice using a Sequenom MassARRAY iPLEX assay (Sequenom Inc, San Diego, CA, USA). Moreover, 500 phenotyped animals of the second filial generation (F2) of HABxLAB intercross mice^24^ were genotyped, allowing an association study of freely segregating alleles with an anxiety-related phenotype, in addition 80 CD-1 mice were included in the analysis.

Primer pairs and single “extension” primers were designed for each SNP in a way that every allele could be genotyped in the same iPLEX-reaction, using a scoring algorithm provided by the manufacturer based on the flanking 100 bp of sequence on both sides of each SNP. Genomic DNA (50 ng/µl) was transferred to 384 well reaction plates. In a primary reaction, the fragments between the corresponding primer pairs were amplified. The remaining dNTPs were dephosphorylated to avoid further binding. In the following iPLEX reaction, the ‘extension’ primers were extended by only one nucleotide complementary to the respective allele. Like the “extension” primer also the nucleotides had a distinct mass. The scoring software provided by the manufacturer was used to calculate the genotypes based on the mass differences.

### In silico analysis for gene regulatory regions

To identify regions prone to regulation by transcription factors, the PROMO database (http://alggen.lsi.upc.es/cgi_bin/promo_v3/promo/promoinit.cgi?dirDB = TF_8.3)^25,26^ was utiliized for potential transcription factor-binding sites on the *Tmem132d* promoter region. After identification of putative transcription factors, their validation was carried out as described in Supplementary Data S1. Genomic coordinates are based on genome builds Mm10 (GRCm38, EnsEMBL release 68) for mice.

### Dual luciferase assays

To examine the role of the identified polymorphisms ~600 bp of the putative promoter region was cloned into pGL3-basic luciferase vectors and transfected into mouse Neuro-2a cells using Exgen 500 in vitro reagent (Thermofisher Scientific, Darmstadt, Germany; Table 3 for primer list). The *Tmem132d* promoter constructs were co-transfected with a pRK5-Gaussia-KDEL vector to normalize transfection efficiency and a SV40-pGL3 vector was used as a positive control. The cells were lysed 48 h post-transfection and processed in a dual luciferase assay as described in^27^.

**Table 3:** Primers used for cloning *Tmem132d* promoter fragments into the luciferase vector

Primer	Orientation	Primer sequence (5’ → 3’)
RVprimer3	Forward	CTAGCAAAATAGGCTGTCCC
GLprimer2	Reverse	CTTTATGTTTTTGGCGTCTTCCA
Tmem132d	Forward	GCAGGTACCCAAGGCTCTGCGGAGCAGTG
Tmem132d	Reverse	GCTAGCAATTTCTCTCTCTTCCTCTCTCCC
Tmem132dreverse310 A sub	Forward	GTTAGGGGTTCCTGAACTGTCCTTGCCTGAAG
Tmem132dreverse310 A sub	Reverse	CTTCAGGCAAGGACAGTTCAGGAACCCCTAAC
Tmem132dreverse310 del	Forward	GACGGTACCTCAGGGACAGGAATTTGAGG
Tmem132dreverse519 A sub	Forward	GTGTGAGTTCGCCTTAGATACCCTGGAAGG
Tmem132dreverse519 A sub	Reverse	CCTTCCAGGGTATCTAAGGCGAACTCACAC

### Oligonucleotide pull-down assay

In order to confirm the functionality of SNP in the *Tmem132d* promoter region (−310 and −519), we performed oligonucleotide pull-down assays to assess binding of POLR2A.

Annealing of oligos was carried out by mixing equal amounts of 5′-biotinylated forward oligonucleotides containing either the HAB or LAB sequence variant and respective non-biotinylated strands (Table 4) along with 10× NEB buffer 2. The reaction mixture was incubated for 4 min at 95 °C, followed by 10 min at 70 °C and finally left on the benchtop for 4–5 h, which allowed annealing of the oligonucleotides. Then, streptavidin beads (GE Healthcare Europe GmbH, Freiburg, Germany) were washed with ice-cold PBS and incubated overnight with the annealed 5′-biotinylated oligonucleotides.

**Table 4:** Primers used for the oligonucleotide pull-down assay

Primer	Orientation	Primer sequence (5’ → 3’)
Tmem132dreverseLABreverse519reverseBiotin	Forward	GTTCGCCTTAGATACCCT
Tmem132dreverseLABreverse519	Reverse	AGGGTATCTAAGGCGAAC
Tmem132dreverseLABreverse310reverseBiotin	Forward	GGTTCCTGAACTGTCCTT
Tmem132dreverseLABreverse310	Reverse	AAGGACAGTTCAGGAACC
Tmem132dreverseHABreverse519reverseBiotin	Forward	GTTCGCCTTGGATACCCT
Tmem132dreverseHABreverse519	Reverse	AGGGTATCCAAGGCGAAC
Tmem132dreverseHABreverse310reverseBiotin	Forward	GGTTCCTGAGCTGTCCTT
Tmem132dreverseHABreverse310	Reverse	AAGGACAGCTCAGGAACC

Nuclear extracts from CD-1 mouse brain tissue were prepared using the NE-PER nuclear and cytoplasmic extraction reagent (Thermofisher Scientific), precleared using streptavidin beads and protein concentration estimated using the Pierce BCA protein assay kit (Thermofisher Scientific). Then, 300 µg of nuclear proteins were incubated with the biotinylated double stranded-streptavidin sepharose complex for 4 h on a rotating shaker, washed with RIPA buffer, heated at 95 °C in sample buffer and separated on 10% SDS-PAGE. The separated proteins were blotted on a nitrocellulose membrane, non-specific binding blocked using 5% milk-TBST and incubated overnight with a polyclonal POLR2A antibody (sc-899×, Santa Cruz Biotechnology, Inc. Frankfurt am Main, Germany) at 1:150 dilution in 2.5% milk in TBST. Subsequently, the blot was washed 3× TBST for 10 min each and incubated for 2 h at room temperature with anti-rabbit HRP-conjugated secondary antibody (1:1000) diluted in 2.5% milk/TBST. Finally, the blot was washed again with 3×TBST for 10 min each and proteins were visualized via chemiluminescence (Western Lighting, Perkin Elmer, Frankfurt am Main, Germany). The protein loading control was carried out by subjecting the blot to colloidal silver staining^28^.

### Adeno-associated virus (AAV) mediated overexpression of *Tmem132d*

To confirm the role of *Tmem132d* in anxiety-related behavior, its corresponding cDNA was amplified from pcDNA 3.1/V5- m-MOLT (kindly provided by Prof. Tomoko Yonezawa, Okayama University Graduate School of Medicine, Japan) using the primers described in Table 5. The *Tmem132d* cDNA sequence was sequenced and confirmed to be identical to the LAB *Tmem132d* cDNA i.e., there were no polymorphisms. Further, we obtained an AAV expression cassette (pAM-CBA-MCS-WPRE2-SpA-containing CMV immediate early enhancer/chicken beta-actin hybrid promoter CBA, multiple cloning site, woodchuck hepatitis virus post-transcriptional regulatory element WPRE2, and SV40 late polyadenylation site). A shorter 0.4Kb neuronal CamKII promoter (to replace 1.1 kb CBA promoter) and an eGFP fluorophore (to confirm viral spread) both derived from Addgene plasmid (# 27226)^29^ were cloned into the AAV expression cassette. Instead of fluorophore, a 3.3 kb of *Tmem132d* cDNA was also cloned into AAV expression cassette and the corresponding plasmid was transfected into Neuro-2a cells to confirm *Tmem132d* overexpression using qPCR. Production of pseudotyped rAAV1/2 mosaic vectors and determination of genomic titers were performed as described in ref. ^30^. Eight-week-old male C57BL/6J mice were anesthetized by inhalation of isoflurane (Baxter Germany GmbH, Unterschleißheim, Germany) and placed on a thermo pad (32 °C) to minimize core body temperature loss. Initially, 2–3 mice were stereotactically injected with a miniscule amount of Nissl’s stain bilaterally into the aCC (+1.5 mm AP, +0.2 mm ML, 1.4 mm DV) from Bregma and then the brain was collected and sectioned to confirm its localization. Another 2–3 mice had AAV-eGFP injected unilaterally (contralateral side internal control with no injection) to confirm viral localization and spread. Subsequently, other mice were randomly assigned using simple randomization to receive either 0.5 µl of 1 × 10^11^ AAV-empty or AAV-*Tmem132d* vector copies/ml via stereotaxic injection bilaterally into the aCC coordinates as described above. The vector delivery was performed using a microinfusion pump at a rate of 0.5 µl/10 min. 2 min after the injection, the needle was carefully retracted and the scalp sutured. Mice were treated with 100 µl Enrofloxacin (Baytril 2.5%; Bayer Vital GmbH, Leverkusen, Germany) s.c. to prevent inflammation and transferred to their home cage for 3 weeks until behavioral testing for anxiety-related behavior. Moreover, *Tmem132d* gene expression was quantified in aCC region harvested from AAV-empty vs. AAV-*Tmem132d* injected mice. AAV-eGFP injected mice were anesthetized with sodium pentobarbital (200 mg/kg) and transcardially perfused with phosphate buffered saline (PBS), 4% paraformaldehyde (PFA) and the isolated whole brain was postfixed overnight in 4% PFA at 4 °C. Subsequently, the brain was rinsed in PBS and immersed in 30% sucrose (to cryoprotect tissue) until brain sinks to the bottom of the tube. Then the brain was snap frozen with n-methyl butane and transferred to −80 °C freezer overnight. Eventually, the brain was embedded in OCT medium (VWR GmbH, Darmstadt, Germany) and cryosectioned into 30 μm thick coronal section, which was imaged for eGFP with a 4× objective on a wide-field fluorescent inverted Leica DM16000 microscope.

**Table 5:** Primers used *Tmem132d* mRNA expression studies and cloning of the *Tmem132d* cDNA into the AAV expression cassette

Primer	Orientation	Primer sequence (5’ → 3’)
Tmem132dreverseexp	Forward	AGGAGCTGGGCATGACCA
Tmem132dreverseexp	Reverse	GAGGTCTGTGATGGTCACCTT
CamkIIp	Forward	CTAGGGTACCCTTGTGGACTAAGTTTGTTCACATCC
CamkIIp	Reverse	CTAGCTCGAGCGGGGATCGGCTCTAGAG
Tmem132d	Forward	CTAGGAATTCGCCACCATGTGCCCATCTGAGATGGGGA
Tmem132d	Reverse	CTAGGAATTCAAGCTTTTATACGTGCTCGTGTAACCTCTCCAT

### Behavioral paradigms

Environmental enrichment (EE): EE paradigm was adapted from Li et al^31^ and Arai et al^32,^ and is composed of a combination of group housing (3 mice per cage) and biologically relevant stimuli such as wood chips and scaffold, plastic insets and tunnel, which along with a bigger home cage allowed mice to interact with their environment and accomplish highly motivated natural behaviors. The EE paradigm consisted of two 14-day periods, called partial and full EE. The partial EE begins from postnatal day (PND) 15–28, when the pups along with their respective dam were transferred for 6 h per day into the bigger cages with different manipulanda. At PND28, pups were weaned and sorted in groups of three and moved for complete EE permanently until PND42.

Unpredictable chronic mild stress (UCMS): The UCMS protocol was adapted from Willner et al^33^ and the timeline was similar to EE. It consisted of alternating mild stressors such as maternal separation, absence of bedding (sawdust), cage tilting (45^0^), mild foot shock (PND15–28) followed by other stressors such as overcrowding, wet bedding, overnight illumination, restraint stress, white noise from PND 29 to 42 to elicit an anxiogenic and pro-depressive phenotype.

The standard HAB and LAB mice were housed under normal conditions without any manipulations (standard housing: SH).

### Elevated-plus maze (EPM) test

The EPM test consists of two open arms (30 × 5 cm) and two closed arms (30 × 5 × 15 cm) joined by a central zone (5 × 5 cm) raised 40 cm above the floor. Light intensity on open and closed arm were 300 lux and < 10 Lux, respectively, while 50 lux in the central zone. The entries and percent time spent on open arm was taken as an index of anxiety-related behavior, while entries and time spent in closed arm serves as an indicator of locomotion during the 5 min test between 8:30 to 11:30 A.M.

### Light–dark box (LDB) test

To examine anxiety-related behavior, the LDB test was carried out for 5 min between 8:30 and 11:30 A.M, which measures percent time spent and distance traveled in the light compartment as well as latency to enter and entries in the light compartment. The LDB test consisted of a dark (16 × 27 × 27 cm) and a light compartment (32 × 27 × 27 cm) illuminated with < 20 Lux and 400 Lux, respectively. EthoVision^®^ XT 5.0 (Noldus Information technology, Wageningen, the Netherlands) software was utilized to track animal movements throughout the test duration.

### qPCR

One cohort of animals were used for RNA extraction, cDNA preparation and real-time PCR from the aCC tissue as described above on a LightCycler 2.0 (Roche Diagnostics, Mannheim, Germany) or 7500 Real-time PCR System (Applied Biosystems, CA, USA). Each experiment was performed in duplicates, standard curves were generated and the relative transcript concentrations were calculated using the 2^(-ΔΔCt)^ method^34^. The primerpair is listed in Table 5.

### Chromatin immunoprecipitation (ChIP) assay

To confirm in vivo POLR2A binding and quantification to the *Tmem132d* promoter A(−310)G SNP in HAB-SH, LAB-SH, HAB-EE, and LAB-UCMS, we utilized ChIP assay using the ChIP-IT express kit (Active Motif, La Hulpe, Belgium) with certain modifications.

aCC tissue (>50 mg) was cross-linked at room temperature for 10 min in ice-cold 1% formaldehyde-phosphate buffered saline (PBS). Then cross-linking was stopped by adding glycine to a final concentration of 0.125 M and incubated at room temperature for 5 min and centrifuged to collect the pellet. Then the pellet was resuspended in lysis buffer and sonicated by applying seven 30 s pulses at high power using standard bioruptor (Diagenode s.a., Liège, Belgium). The sheared chromatin was utilized for immunoprecipitation with POLR2A antibody (sc-899×, Santa Cruz Biotechnology) and protein A/G plus-agarose (sc-2003, Santa Cruz Biotechnology) along with buffers provided in the ChIP-IT express kit. The immunoprecipitated DNA-protein A/G plus agarose complex was subsequently washed, eluted, reverse cross-linked and treated with proteinase K to obtain the enriched DNA fraction as described in the ChIP-IT express kit. The end point quantitative PCR was carried out using primers flanking the A(-310)G SNP region (Table 6) and also mouse positive and negative primers (Active Motif) for POLR2A binding at active and inactive regions.

**Table 6:** Primers used for qPCR end point analysis of ChIP enriched fractions of POLR2A

Primer	Orientation	Primer sequence (5’ → 3’)
Tmem132dreverse310	Forward	ATTTTGGCATCCCAGGCTCA
Tmem132dreverse310	Reverse	AGGTGTGGAAAGGCCATCTG

### Data analysis

Behavioral phenotypic and gene expression differences were analyzed using either the two-tailed Student’s *t* test or one-way ANOVA with post hoc Bonferroni’s corrections using GraphPad Prism software version 7.02 (La Jolla, CA) and significance was accepted at *p* < 0.05. The results are presented as mean ± s.e.m. with animal number and replicates indicated in figures or figure legends. Animal sample size was justified by previously published data^8,35^ or preliminary experiments. All experimental animals with similar behavioral profile (e.g., HABs for standard-housing or environmental enrichment) or wild-type mice were randomly allocated to experimental groups. The distribution of the data was assumed to be normal; however, this was not formally tested. Whenever possible, the experimenter was blind to experimental and/or treatment group.

### Methylation analysis in patients with anxiety disorders

PD patients were recruited at the outpatient unit of the Max Planck Institute of Psychiatry (MPIP) in Munich^15^. All patients included in the current analyses (*N*_total_ = 74, *N*_males_ = 26, *N*_females_ = 48) were Caucasian and were not taking any psychotropic medications. Anxiety symptoms were assessed with the Hamilton Anxiety scale (HAMA)^36^ and severity of panic symptoms was assessed with the Panic and Agoraphobia scale (PAS)^37^. Positive Life Events were assessed using a German modified version^38^ of the Social Readjustment Rating Scale^39^. All subjects provided written informed consent and procedures were approved by the Ethics Committee of the Ludwig Maximilians University, Munich, Germany, in accordance with the Declaration of Helsinki.

### DNA methylation arrays in the MPIP Study

Genomic DNA was extracted from peripheral blood using the Gentra Puregene Blood Kit (Qiagen). DNA integrity and concentration was assessed by NanoDrop 2000 Spectrophotometer (Thermo Scientific) and Quant-iT Picogreen (Invitrogen). Genomic DNA was bisulfite converted using the Zymo EZ-96 DNA Methylation Kit (Zymo Research) and DNA methylation levels were assessed for >480,000 CpG sites using the Illumina HumanMethylation450 BeadChip array. Hybridization and processing was performed according to the instructions of the manufacturer. Quality control of the methylation data included intensity read outs, normalization, cell type composition estimation, β- and M-value calculation and was performed using the Bioconductor R package “minfi” (version 1.10.2). X-chromosome, Y-chromosome, and non-specific binding probes were removed^41^ together with 236 failed probes that were excluded based on a detection p-value larger than 0.01 in >50% of the samples. Probes containing a SNP in the sequence or located close (10 bp from query site) to a SNP, which had a minor allele frequency of ≥ 5% were also removed. The data were then normalized using functional normalization^42^, an extension of quantile normalization included in the R package “minfi”. Batch effects were identified by inspecting the association of the first principal component of the methylation levels with bisulfite conversion plate, well, slide, slide row and array using linear regression and visual inspection of PCA plots using the Bioconductor R package *shinyMethyl* (version 0.99.3). The Empirical Bayes’ (EB) method *ComBat* was used to remove batch effects.

### Gene-environment interaction analysis

To test whether there was an association between *TMEM132D* methylation levels and the number of positive life events experienced depending on previously described risk alleles for PD in the rs7309727 SNP (*N*_TT_ = 38, *N*_CT_ = 36, *N*_CC_ = 0), a linear regression model was fitted with the batch corrected M-values for each of the 73 CpGs located in the *TMEM132D* gene. Gender, age and Houseman-calculated blood cell counts^42^ were included as covariates in all the regression models.

### POLR2A ChIP-seq of *TMEM132D* gene

The RNA Pol II ChIP-seq data have been obtained with K562 chronic myelogenous leukemia^43^ and GM78^44^ lymphoblastoid cell lines by Michael Snyder lab at Yale University (GEO accession number GSE13008 and GSE30399, respectively). These data are made publicly available by ENCyclopedia Of DNA Elements (ENCODE) analysis on UCSC genome browser (http://genome.ucsc.edu/)^45–47^, and were utilized to view the POLR2A ChIP-seq data for *TMEM132D* gene on the human feb. 2009 (GRCh37/hg19) Assembly.

## Results

### Polymorphisms in the *Tmem132d* gene

**Fig. 1:**
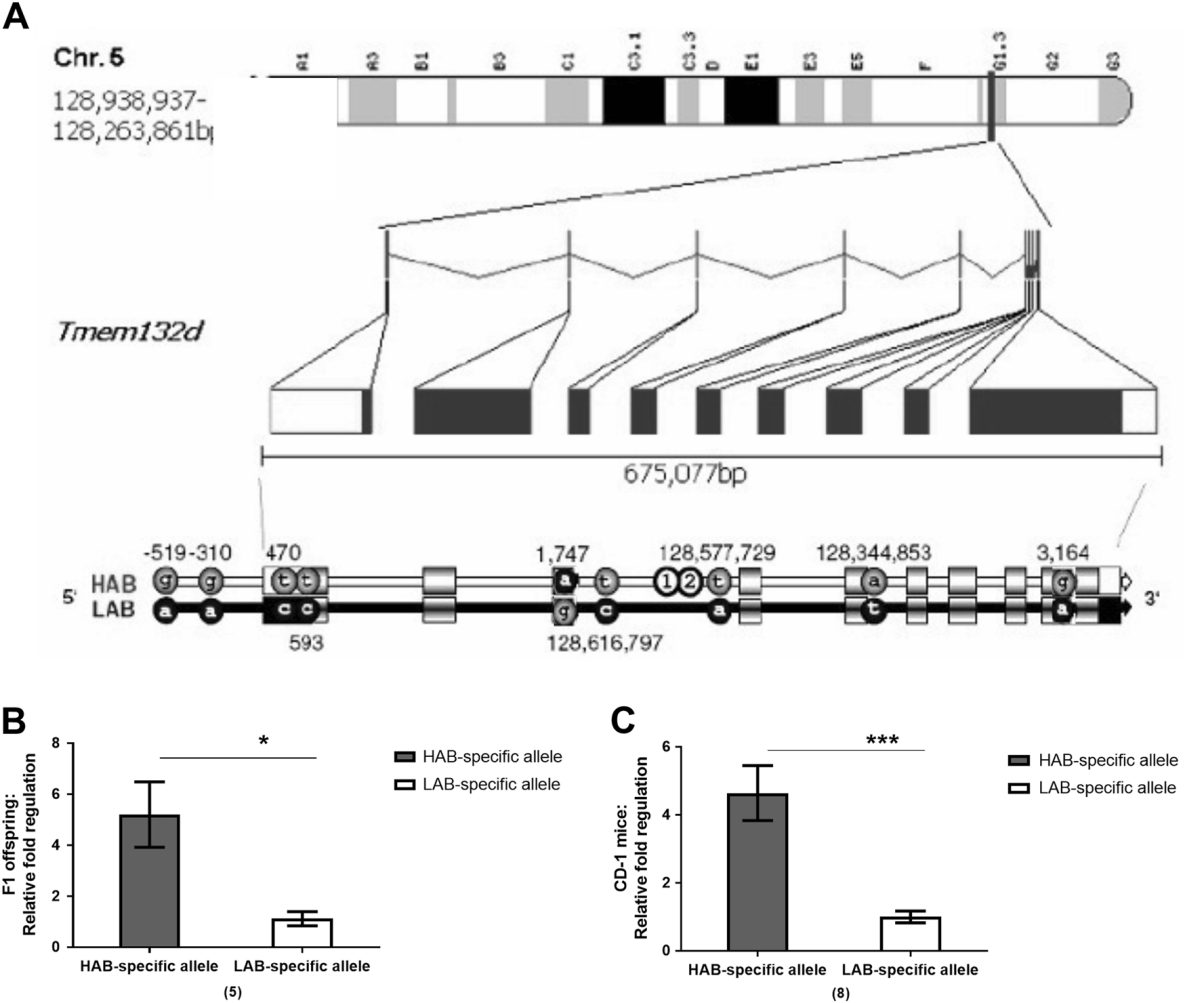
**a** Transmembrane protein 132D *(Tmem132d)* gene sequence of HAB vs. LAB mice. Polymorphic sites are indicated with minus sign for promoter positions in bp, with positions in the coding sequence from transcription start in the spliced mRNA (in bp) or with chromosomal positions for single-nucleotide polymorphisms in introns (in bp). Exons and untranslated regions (UTRs) are indicated by boxes (exons shaded, UTRs completely filled black or white). Unfilled circles with numbers ‘1’ and ‘2’ refer to two deletions in HAB mice. For ‘1’ at position 128,577,978 bp (deletion of CAAA) and ‘2’ at position 128,577,906 (deletion of ACA). Allele-specific transcription assay in HABxLAB cross-mated F1 **b** and CD-1 **c** mice heterozygous for rs36596918. The data are presented as mean ± SEM and numbers in parentheses indicate the group size; ***p < 0.0001; *p < 0.05

To understand the differential expression of the *Tmem132d* mRNA in the aCC of HAB vs. LAB, its putative promoter region up to 1000 bp and coding regions were sequenced. Sequencing *Tmem132d*, several polymorphic loci could be identified, among them, two SNPs in the promoter of the gene at positions A(−519)G and rs233264624 (A(−310)G). Four further SNPs were identified located in exons, with C(470)T and C(593)T in the 5’ untranslated region and G(1,747)A in exon 3 (also known as rs36596918) and A(3,164)G in exon 9 (rs13478518) as part of the protein coding sequence. Furthermore, three SNPs and two deletions were discovered in introns, among them C(128,616,797)T (rs13478520), A(128,577,729)T (rs6387514), Δ(128,577,906-128,577,903) (rs237178791) and Δ(128,577,978-128,577,974) in the third and T(128,344,853)A in the fifth intron (Fig. 1a and Table 7).

**Table 7:** Overview of the identified polymorphisms in *Tmem132d* in HAB vs. LAB mice including the flanking sequences. The HAB polymorphisms are underlined

Genetic region	Polymorphism	sequence: 5’ → 3’
promoter region	A(−519)G	TTCCTCTCTGAATCCCCACAACATCTGCTTGTTTCCAAGGCTCTGCGGAGCAGTGAGGAGGGCTCCGGCTCGGGACACTAGCATGGGTATTATGTATCTTGGTGTGAGTTCGCCTT[A/G]GATACCCTGGAAGGCATGAGTGGAGAGCTGTACCTATTAGCTTTTCAATGAAATATCTTCCCTAATGCCAATTCCCTAATGCCCAAAGGCCAGCGGGGAGCCAGGATGGAAATCTTT
	A(−310)G rs233264624	CGGGGAGCCAGGATGGAAATCTTTCAGGGACAGGAATTTGAGGGTCATTTTGGCATCCCAGGCTCACAATCTAGAGTTCACCGGAAACACTTCAGCCTATGTTAGGGGTTCCTGA[A/G]CTGTCCTTGCCTGAAGGCCGCCTATAGTCAGAAGCTTTAAGAAGGCAGGGCAGATGGCCTTTCCACACCTGTTGTGAGCGTGGACATCCACGCTGGTTCTCGTGCGCGCTCCTCGGGGCTTGCGGGGTGTCCATAACCTC
exon 1 5’ untranslated region	C(470)T	GCAGCCCCGCAGCCGCCCGCGCGCGCTAGCCCACCGCAGCCTCCCAGCCGGAGTCCTCAGAGCGCTCTCCATGCTTGGGGACGCAACACCGGAGACCCCCCTTGTGGAGTGAAATTCAATTCACGAGC[C/T]AGTCCTCAGCGCAGTCGAAAGACTGAGGTGAGCCTCGGCCTGCGCTGCGGGACCCCGGAGCCTGAGCGGCCTGGCTATGGACCTGTGGGGACCCGGCACAGGGCGGCCCTGCTGCTCGGG
	C(593)T	TCCTCAGCGCAGTCGAAAGACTGAGGTGAGCCTCGGCCTGCGCTGCGGGACCCCGGAGCCTGAGCGGCCTGGCTATGGACCTGTGGGGACCCGGCACAGGGCGGCCCTGCTGCTCGGGTC[C/T]CTAGCCAGATGCGTCTTGGTCATCCAGCTCAGCTTCTCTTCTCCGGGGATCTGGCGACATCCCTTCTTCAGCCAGAGGACACCACGGAGGAGCAGAGGACCCGCGCTCCAAGTCTCCAGGATGTGCCCAT
exon 3	G(321607)A rs36596918	ACCACTGCTGCCCGGGGACTTCCCCAGGGCTATCTCAAACATGCACTTCCTGTCTCCAACAGGGCCAAGGTTAAGAAAGGAGTGAGCATTGTTGGGGTGAGAGCCAGCAGCTCTTCAATCTGGGATGTCA[G/A]ACAGAGCACTGAGTACACTGGGAAATATGCTCCGGCCGTCATCGTTTGTCAGAAGAAATCTGCTGGCTCCGAAAAGAGGTAAGTCCCACCTGGCCTGTGCTGTGCTCAGACTCTCTGCCATGGTGGCTTC
intron 3	Δ(360972-6) rs237178791	GTAACAAGGACTGGGAAAGATGTGGACAGACACTCACAAGAAGCTGATAGAATGTAATTTAGTCCAACTGCTGCCATGGGTTTGGTATGTTGGGTTCTCAAAACAAACAAACAAACAAA[CAAA]ACTAACAAAACACCAAACCAAGACAACCCAAACAAACCATCATCATCAACAACAACAACAACAACA[ACA]GCCTTGAAATCAACTTTGAGTATATGCATACTGTGTTTTCTGTAGGCATCTGAGAGAACAGAATTGCTAATCAGAAAAGCAGCAAA
	C(322141)T rs13478520	TTGCCCAGCTGTGAATTGCCTGTTTCTAATTTGGTGGCTATCAGAAGCAGATCCGGGTTTTAATTTTTCTCCCCGGGGTGATTCACGATCGCCTGTAATTCCACAGCGGG[C/T]GGCCCACTGTTCCATCAGAAAGTGCCCCCTGCACAAATACAATCAAATGCAGTATGTTGACATCAGCAGCGCCGTAAAGGTGGGCTGCACCCTGCCTGTAGAAGAGCGAG
	A(361209)T rs6387514	AACAGAATTGCTAATCAGAAAAGCAGCAAAACATGATTATGAGACGTGATAGGCCCATGTGCTATCATGTGTGGAGTGTGCAGGCTTTAAGCATGCAGACATTCCTACCATCGGCGCCTG[A/T]AATCCTAAGTGGTGGGTGTATAACTAGATGCTCTCTGCGCAGAGTTTAGCAGTCTTATGTTCCCACACTCTCCAGACCAGATTCTTTTTTTGATGTCCCTTACTCTGTACAGAAAAATCCCCCTCTTGTT
intron 5	T(594085)A	TGACGAAGACGTGGTTAAGGCAAGTGGATGGTTCCTCGCCCATTAGGAAGCCCATAGTTACACGGGCATGGGTGTTTATCCCCAGCTGAGATCTGTGGGGTCTGCAGGGTTCCTCCCATTCTGGTTTTCT[T/A]GCAATTTAAATCAGGAGTGTCTCCCACCCCAACGCCAAATAGGATATACACTGCTATAACCCATGCCTGGGCTGTGAGACATTTCTTCTGTGCCT
exon 9	A(673963)G rs13478518	AATCTTGCCAGAAGTCTAAGCGGAAGAGCGTGCTGGCTGTGGGAACAGCCAGCATCAAGGTTAAATTTGGACAGAATGATGCAAACCCCAACAGCAGTGAGAGTGGACACCTTGGGGCTGGGCTTCATGT[A/G]GAGAATATCAATGACAGGAGGTCCAAAAAGCCTTTTCAGGAATGGGGGAGCCCCGAGGGGCCATTCTACAGCAGCTCATCCATGGGGCTCATGGAGGGATGGGGCAGTACCACCAAGAGGCCAACTTTC

### Allele-specific transcription of *Tmem132d*

The analysis of allele-specific transcription based on G(1,747)A (rs36596918) revealed a significantly higher transcription of the HAB-specific allele with both alleles present in the same cell. This held true for HABxLAB F1 offspring and CD-1 mice heterozygous for *Tmem132d* (*p < 0.05 and ***p < 0.001, respectively, Figs. 1b, c).

### Genotype–phenotype associations (Sequenom assay)

In the HAB-LAB-derived F2 mice^24^, all SNPs discovered in HAB vs. LAB mice and analyzed in *Tmem132d* displayed similar distribution patterns, as the genomic locus is not larger than 800 kbp, however, some recombination has occurred over two breeding generations. Altogether, 10 recombination events have been seen in 501 mice over two generations, thus resulting in a recombination rate of 5 per generation or 1/generation and 100 individuals, i.e., a recombination rate of 1/100 within the given locus. Thus, the association of *Tmem132d* genotypes with anxiety-related behavior, as described^15^ remains significant for all SNPs in *Tmem132d* (*p* < 0.01). The additional analysis of 80 unrelated CD-1 mice revealed, that rs13478518 (the SNP in exon 9) showed higher variation, as the other SNPs did, with the HAB-specific allele being the minor allele (minor allele frequency—MAF = 0.39). For the other SNPs described for *Tmem132d* in CD-1 mice, the HAB-specific variations were also the minor alleles, with MAF = 0.08. However, due to the small sample size, genotype–phenotype associations did not reveal any significant association in CD-1 mice.

### Promoter SNPs are sufficient to explain the higher *Tmem132d* mRNA expression

**Fig. 2:**
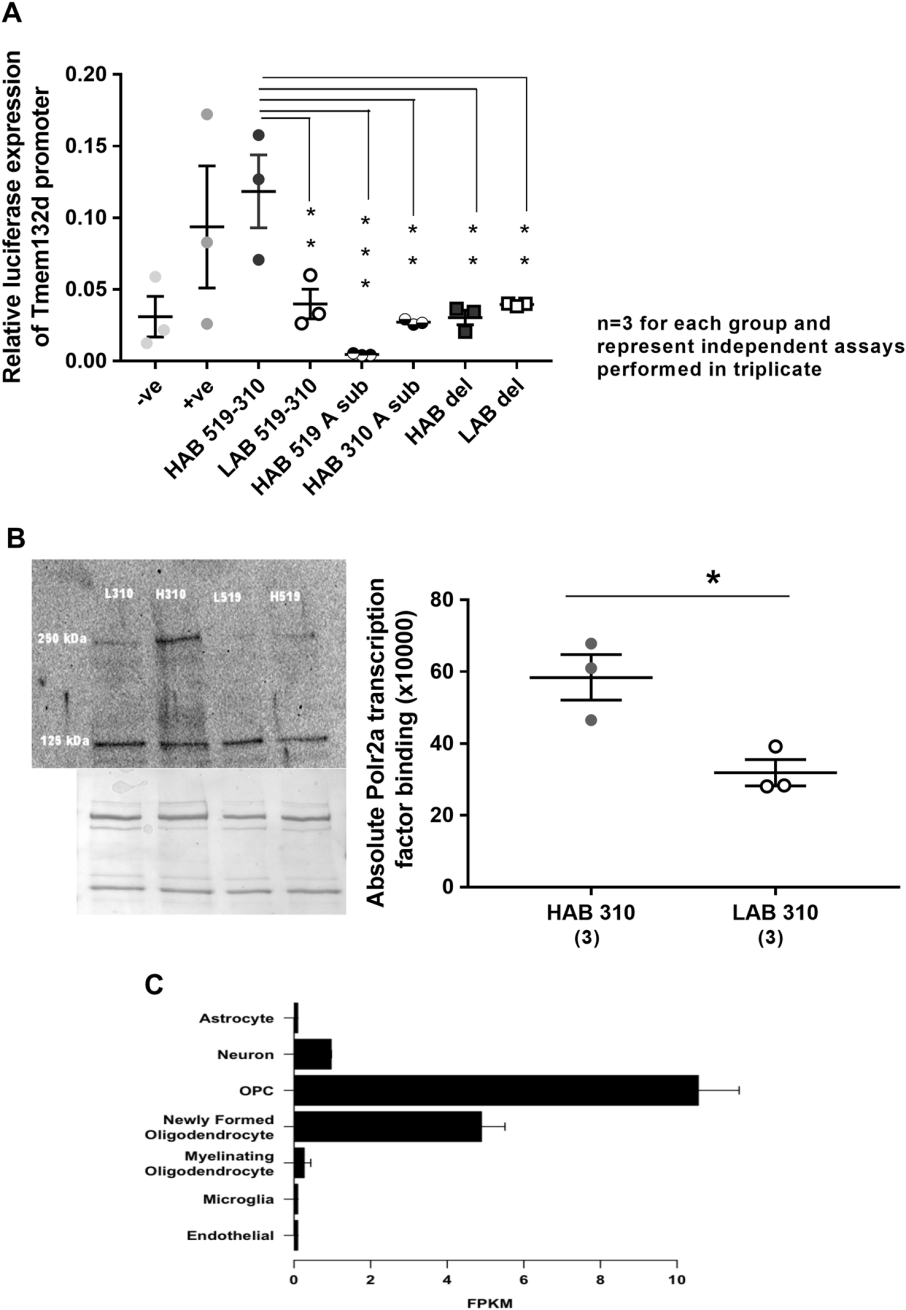
**a** Dual luciferase assay with HAB vs. LAB *Tmem132d* promoter constructs. Data are shown as mean ± SEM; **p < 0.01; ***p < 0.001; in comparison with HAB-specific SNPs (−519)G and (−310)G. Firefly luciferase data were normalized to Gaussia activities and are presented as relative expression ± SEM of three independent experiments performed in triplicate. Negative (−ve) and positive (+ve) controls represent pGL3 basic and SV40-pGL3 vectors, respectively. **b** Semi-quantitative western blots and representative image (upper) for POLR2A transcription factor binding to rs233264624 with the HAB-specific (−310)G vs. LAB-specific A(−310) along with corresponding colloidal silver staining (lower), which served as loading control. The data are shown as means ± SEM, *p < 0.05 (*n* = 3 for each group and represent independent assays performed in triplicate). **c** RNA- seq data showing *Tmem132d* differential expression calculated as the Fragments Per Kilobase of transcript per Million mapped reads (FPKM) of a given cell type divided by the average FPKM of all other cell types (source: Zhang et al 2014)^14^

The LAB vs. HAB *Tmem132d* promoter carrying the A(−519)G and A(−310)G SNPs, respectively were cloned into pGL3-basic vector and promoter activity was measured using dual luciferase assay. The whole HAB *Tmem132d* promoter constructs containing guanine nucleotide(s) at the positions (−519) and (−310) exhibited higher luciferase expression (**p < 0.01; Fig. 2a) compared to corresponding LAB constructs, suggesting increased promoter activity. These data are in line with the *in vivo* findings of higher *Tmem132d* mRNA expression in HAB-SH compared to LAB-SH. To dissect the individual contribution of SNPs, mutation of the guanine nucleotide to adenine at (−519) or (−310) caused a significant decrease in promoter activity (***p < 0.001 and **p < 0.01), respectively in comparison to whole HAB-sequence-based constructs. This indicates that both guanine residues are important for the higher HAB promoter activity. Meanwhile, deletion of the A nucleotide at position (−519) in the LAB construct did not have any effect on its promoter activity. While, the deletion of the G nucleotide at (−519) in the HAB construct again led to significant decrease in promoter activity (**p < 0.01; Fig. 2a), thus proving the importance of the G nucleotide at (−519) for the observed expression.

The discovery of a CpG island in the upstream *Tmem132d* promoter prompted us to do bisulfite sequencing (Table 8). There was no difference in total DNA methylation between HAB/LAB basal *Tmem132d* promoter regions. Supplementary Data S2 illustrates these findings. Selective or complete methylation of *Tmem132d* promoter (Table 9) did not alter its promoter activity (data not shown).

**Table 8:** Primers used for bisulfite sequencing of the *Tmem132d* gene in HAB vs. LAB mice

Primer	Orientation	Primer sequence (5’ → 3’)
M13 tailed Bisulfite sequencing		
Tmem132d	Forward	TGTAAAACGACGGCCAGTGGAGTGATGTTGGGTTTTTTTT
Tmem132d	Reverse	CAGGAAACAGCTATGACCTTTTAAACCCCACCCTTCTAAA
M13 tailed genomic DNA sequencing		
Tmem132d	Forward	TGTAAAACGACGGCCAGTGGAGTGATGCTGGGTTTCCTCT
Tmem132d	Reverse	CAGGAAACAGCTATGACCTTTTAAGCCCCACCCTTCTGGA

**Table 9:** Primers used for in vitro methylation studies

Primer	Orientation	Primer sequence (5’ → 3’)
LAB Tmem132d	Forward	GTATTATGTATCTTGGTGTGAGTT[5MedC]GCCTTAGATACCCTGGAAGGCATG
LAB Tmem132d	Reverse	CATGCCTTCCAGGGTATCTAAGG[5MedC]GAACTCACACCAAGATACATAATAC
HAB Tmem132d	Forward	GTATTATGTATCTTGGTGTGAGTT[5MedC]GCCTTGGATACCCTGGAAGGCATG
HAB Tmem132d	Reverse	CATGCCTTCCAGGGTATCCAAGG[5MedC]GAACTCACACCAAGATACATAATAC

### Binding of putative transcription factor at regulatory regions of *Tmem132d*

The oligonucleotide pull-down assay indicated that binding of the transcription factor POLR2A occurred at significantly higher levels to the HAB-locus containing the G nucleotide at position (−310) as compared to corresponding LAB-specific locus (*p* < 0.05; Fig. 2b). The band size was, as expected, approximately 250 kDa in size (Fig. 2b). In addition, there was an unknown band at ~125 kDa of equal intensity in all samples (Fig. 2b).

### Higher expression of *Tmem132d* in the aCC causes increased anxiety-related behavior

**Fig. 3:**
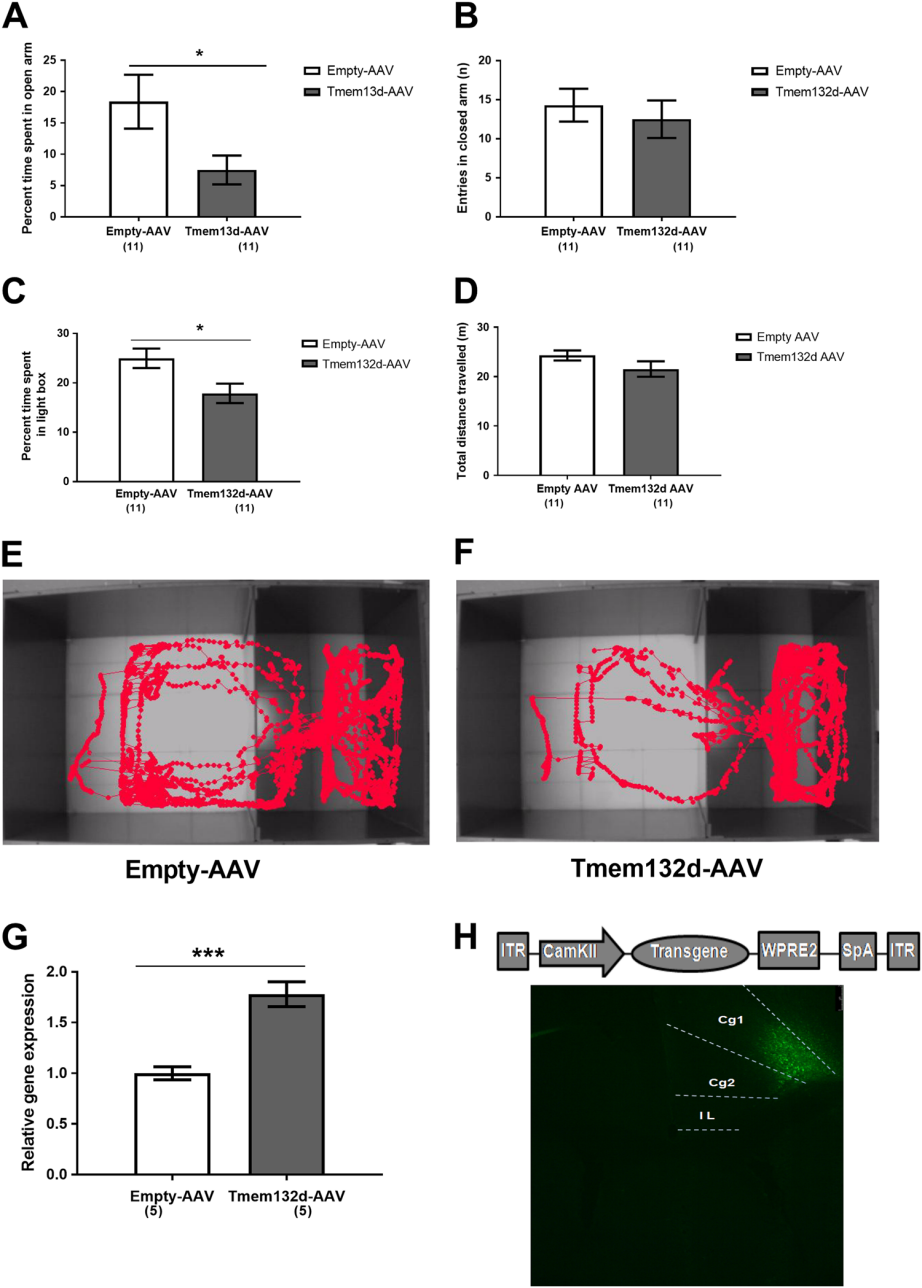
Adeno-associated virus mediated overexpression of *Tmem132d* or eGFP in the anterior cingulate cortex of C57BL/6 J mice. Percent time spent in open arm (**a**), and entries in closed arm (**b**) on the elevated-plus maze. Percent time spent in light compartment (**c**), and distance traveled (**d**) in the light–dark box test. Representative image of animal movement tracks of empty-AAV (**e**) vs. *Tmem132d*-AAV (**f**) injected C57BL/6 J mice. (**g**) Validation of *Tmem132d* mRNA expression in the anterior cingulate cortex of empty-AAV vs. *Tmem132d*-AAV injected C57BL/6 J mice. The data are shown as means ± SEM, numbers in parentheses indicate the group size; *p < 0.05, ***p < 0.001. (**h**) Representative image showing enhanced green fluorescent protein expression in anterior cingulate cortex (Cg1, Cg2) of C57BL6/J mice

Overexpression of the *Tmem132d* gene using an AAV-mediated system in the aCC caused C57BL/6 J mice to spend significantly lower percent time in open arm of EPM and light compartment of LDB test compared to mice injected with empty control AAV (p < 0.05; Fig. 3a, c, respectively). Furthermore, there was no difference in locomotion between the two groups of mice (Fig. 3b, d). Animals injected with *Tmem132d*-AAV have sparse tracks in the light compartment compared to those injected with empty-AAV (Fig. 3e, f). We could also confirm higher *Tmem132d* mRNA in the *Tmem132d*-AAV injected mice relative to empty AAV (*p* < 0.001; Fig. 3g). AAV-eGFP injected into aCC could confirm viral spread (Fig. 3h).

### Bidirectional nature of anxiety phenotype and plasticity of the *Tmem132d* gene

**Fig. 4:**
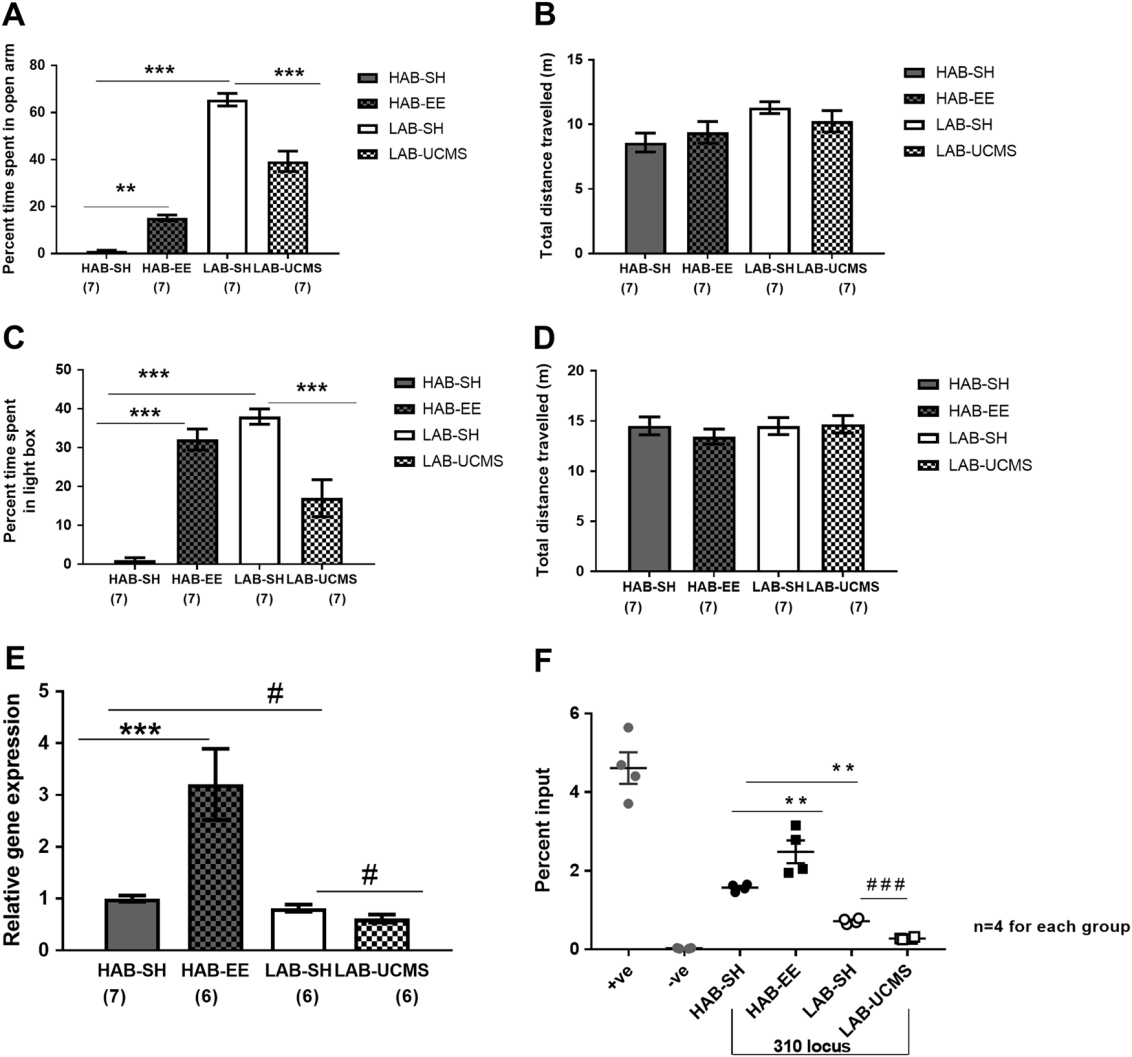
Effect of environmental enrichment (EE) and unpredictable chronic mild stress (UCMS) on HAB and LAB mice, respectively. Percent time spent in open arm (**a**), and total distance traveled (**b**) on the elevated-plus maze. Percent time spent in light compartment (**c**), and total distance traveled (**d**) in the light–dark box test. **e** Relative *Tmem132d* mRNA expression in HAB-SH, HAB-EE, LAB-SH and LAB-UCMS in the anterior cingulate cortex. Data are shown as means ± SEM, numbers in parentheses indicate the group size; ^#^ *p* > 0.05, **p < 0.01, ***p < 0.001. **f** Chromatin immunoprecipitation assay for POLR2A binding to (rs233264624) A(−310)G locus of HAB-SH/ HAB-EE/ LAB-SH/ and LAB-UCMS. Negative (−ve) and positive (+ve) controls represent occupancy of POLR2A at the active and inactive chromatin marks. The data are shown as means ± SEM with *n* = 4 for each group. **p < 0.01, ^###^ *p* < 0.001 (*t* test)

One-way ANOVA revealed overall statistically significant difference in both percent time spent on open arm of EPM (*F*_3,24_ = 117.3; *p* < 0.001: Fig. 4a) and light compartment of LDB (*F*_3,24_ = 31.72; *p* < 0.001: Fig. 4c), respectively. Furthermore, Bonferroni’s post hoc test uncovered that HAB-EE spent significantly higher percent time on open arm of EPM and light compartment of LDB in comparison to HAB-SH (Fig. 4a, c, respectively). While, LAB-UCMS spent significantly lower percent time on open arm of EPM and light compartment of LDB (Fig. 4a, c, respectively). Moreover, there was no difference in total distance traveled in both EPM and LDB test amongst the four groups (Fig. 4b, d, respectively).

One-way ANOVA also revealed an overall significant difference (*F*_3,21_ = 12.69; *p* < 0.001; Fig. 4e) in *Tmem132d* gene expression in aCC with Bonferroni’s post hoc test uncovering significantly enhanced expression in HAB-EE compared to HAB-SH (*p* < 0.001). Moreover, unpaired *t* test revealed a trend towards significance with higher gene expression in HAB-SH compared to LAB-SH (*t*_11_ = 2.047; *p* = 0.0653), which validates earlier studies^15^. We also observed a trend towards significantly decreased gene expression in LAB-UCMS compared to LAB-SH (*t*_10_ = 1.953; *p* = 0.0794).

### ChIP to assess in vivo POLR2A binding

One-way ANOVA with Bonferroni’s correction of ChIP data confirmed a significantly higher binding of POLR2A at the HAB-specific guanine nucleotide at (−310) locus compared to the corresponding LAB-specific adenine nucleotide (*p* < 0.01; Fig. 4f). In addition, we found enhanced binding of POLR2A to the HAB-EE (−310) locus compared to respective HAB-SH region (*p* < 0.01). Student’s *t* test revealed lower POLR2A binding in the LAB-UCMS (−310) locus in comparison to corresponding LAB-SH locus (*p* < 0.001). In a separate set of immunoprecipitated DNA from mouse brain tissue, higher occupancy of POLR2A was detected in the promoter region of the housekeeping gene beta-actin and sparse POLR2A binding in a genetic desert on chromosome 6. Thus, both positive and negative control primers (Active motif) confirmed their role as transcriptionally active and inactive chromatin marks, respectively.

### *TMEM132D* methylation is associated with Positive Life Events and rs7309727 SNP variants in anxiety disorder patients

**Fig. 5:**
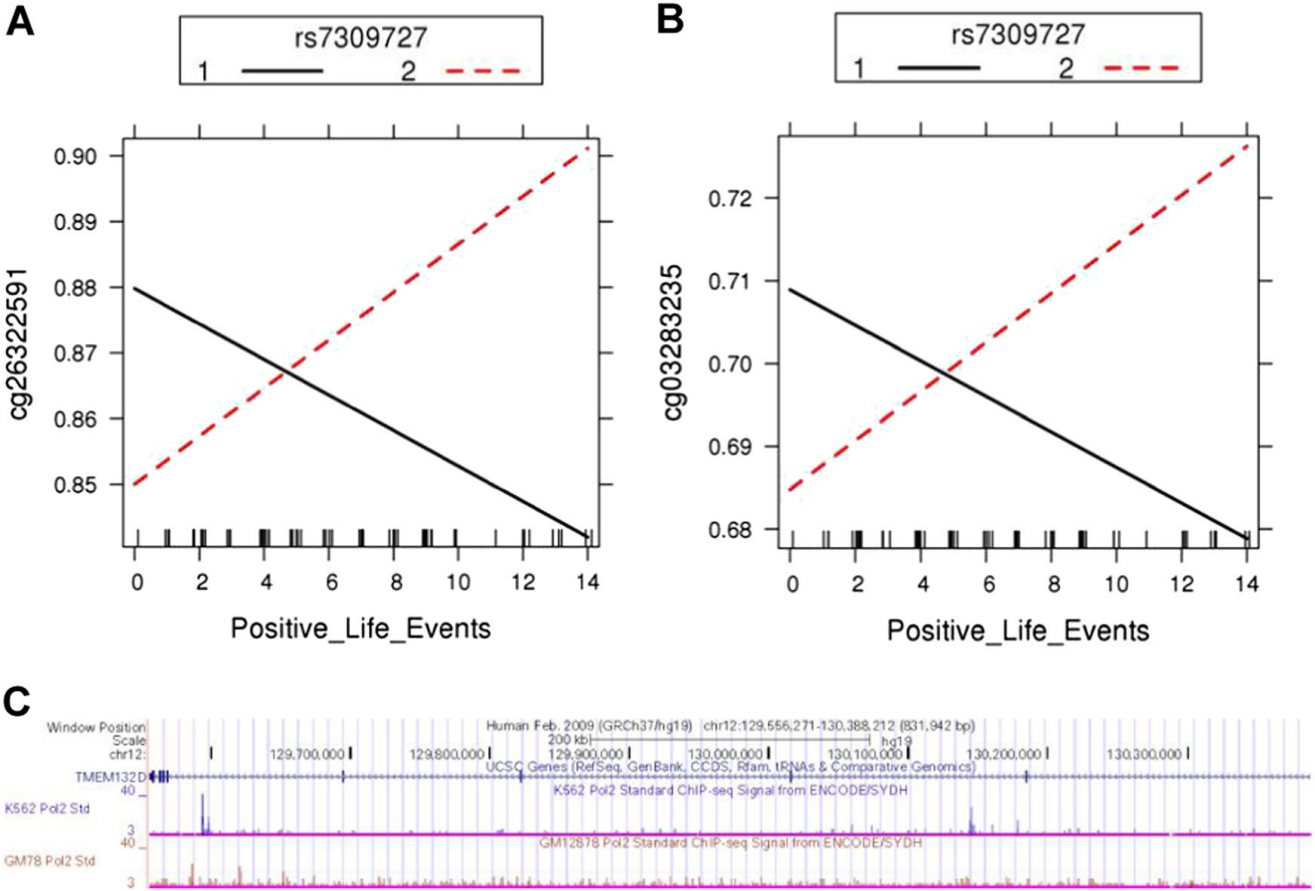
Interaction plot of (**a**) cg26322591 and (**b**) cg03283235 with Positive Life Events depending on rs7309727 genotypes (1 = CT, 2 = TT). *X*-axis: methylation levels expressed as β-values. *Y*-axis: Number of positive life events experienced. (**c**) Illustration of the RNA polymerase II ChIP-seq data from ENCODE project for K562 and GM78 human cell line showing POLR2A enrichment across *TMEM132D* gene body in UCSC genome browser on Human Feb. 2009 (GRCh37/hg19) Assembly

In previous studies, rs7309727 was found to be associated with PD with higher risk for T allele carriers^16^. Two CpGs (cg26322591 and cg03283235) located in the *TMEM132D* gene were significantly hypermethylated (*P*_FDR_ = 0.0296) in T homozygotes anxiety patients experiencing a higher number of positive life events while CT heterozygotes showed lower methylation levels with a higher number of positive life events experienced. We also performed the analysis for association with negative life events and did not find any significant results (Fig. 5a, b).

### POLR2A enrichment across *TMEM132D* gene body

There was substantial enrichment of POLR2A across *TMEM132D* gene, including the intronic regions in K562 and GM78 cell lines (Fig. 5c).

## Discussion

Selective breeding of mice over several generations causes genetic variation associated with the trait anxiety to be represented at a higher frequency, leading to homozygosity at loci conferring this trait^48^. Anxiety disorders are spread across a continuum in a form of normal distribution with HAB and LAB denoting the two opposite extreme ends. The *TMEM132D* gene was first identified through genome wide association study in PD patients, in which intronic SNPs were associated with anticipatory anxiety in these patients as well as to severity of anxiety symptoms in patients with major depression or panic attacks, but not to the severity of depressive symptoms^15^. This suggests that the genetic variants are specific for the severity of experienced anxiety but not restricted to any particular disorder^15^. One of the associated variants showed regulatory function associating with higher *Tmem132d* mRNA expression in the frontal cortex of postmortem brain tissue, suggesting their functional role in anxiety pathophysiology^15^. Notably, a recently published study investigating quantitative trait loci in fear conditioning in mice revealed *Tmem132d* as suggestive locus for fear acquisition supporting its involvement in anxiety circuits^20^. Concurrently, in the HAB/LAB mouse model, *Tmem132d* was identified through microarray analysis and later confirmed independently through qPCR to be higher expressed in the aCC of HAB-SH compared to LAB-SH mice and intermediate levels in CD-1 mice^15^. There was no such differential expression observed in the central, basolateral amygdala, hypothalamic paraventricular nucleus, and dentate gyrus of HAB/LAB mice^15^.

To study the genetic underpinnings behind this differential expression, we sequenced *Tmem132d* and found two SNPs A(−310)G and A(−519)G in the putative promoter of the gene. We observed two guanine (G) residues in the HAB *Tmem132d* promoter in place of the adenine (A) residues in the corresponding LAB locus. We also observed a SNP in the 3rd exon (rs36596918) that causes substitution from arginine to lysine, however, the functional significance of this SNP is currently unknown. Also, another SNP in the 9th exon (rs13478518), part of untranslated region, was found to co-segregate with anxiety-related behavior in an F2 panel, independent of depression-like behavior and locomotor activity^15^, advocating a causal role of the *Tmem132d* gene in anxiety-related behavior.

The rs36596918 was utilized as allele-specific tag to quantify the HAB and LAB allele-specific contribution to *Tmem132d* expression in cross-mated (HABxLAB) F1 offspring and CD-1 mice and were found to have higher HAB allele-specific expression in comparison to LAB allele-specific. Similar data were observed with F1 offspring and CD-1 mice using rs13478518 as allele-specific tag (data not shown). To dissect the functional importance of promoter SNPs, we amplified and cloned the HAB/LAB *Tmem132d* promoters in gene reporter (luciferase) vectors. The resultant assay revealed higher luciferase expression, suggesting that the two SNPs indeed caused higher *Tmem132d* mRNA expression. Furthermore, to understand the putative transcription factor that might modulate this differential expression, we carried out in silico analysis, which suggested binding of nuclear factor I/C (NFI/C; CCAAT-box binding transcription factor) at the HAB -specific locus (−519)G and general transcription factor II B (GTFIIB) at the proximal (−310)G HAB position, respectively. Overexpression or short hairpin RNA (shRNA) knockdown of NFI/C or GTFIIB did not alter luciferase expression of the HAB/LAB *Tmem132d* promoter (data not shown).

Nonetheless, NFI/C has been shown to be involved in eukaryotic transcription^49^ and colocalizes with RNA polymerase II^50^. Moreover, studies have shown that binding of NFI/C is blocked with CpG methylation resulting in increased promoter activity of Igf2^51^. However, bisulfite sequencing of standard-housed HAB and LAB *Tmem132d* promoter harboring a CpG island did not reveal any difference in total percentage of DNA methylation (Supplementary Data S2). Moreover, selective or complete methylation of HAB/LAB *Tmem132d* promoter region did not alter luciferase expression i.e., promoter activity (data not shown). But it is plausible that DNA methylation might bind at other regulatory regions in the gene body and regulate its expression. It is worth noting that the GTFIIB that binds −310 SNP is involved in the formation of the RNA polymerase II preinitiation complex and aids in initiation of transcription^52,53^. Thus, both the guanine residue in HAB *Tmem132d* promoter seem to interact with RNA polymerase II, and therefore, we carried out oligonucleotide pull-down assay for one of its largest subunit i.e., RNA Polymerase II subunit B1 (POLR2A). We observed significantly higher binding of POLR2A to the HAB-specific (−310)G position compared to corresponding LAB locus, suggesting that the HAB-specific (−310)G variant causes a gain-of-function leading to higher binding of POLR2A and possibly, increase in transcription. Thus, we could first identify one regulatory component in the *Tmem132d* gene, which is relevant for a behavioral phenotype in mice. We utilized four different commercial antibodies to examine TMEM132D protein expression difference in aCC of HAB-SH and LAB-SH along with samples from *Tmem132d* ^(-/-)^ knockout (KO) mice, however, none of them were specific for the target gene (Supplementary Data S3).

We also utilized AAV-mediated overexpression of *Tmem132d* in the aCC of C57BL/6J mice and observed lower percent time spent in open arm of EPM and light compartment of the LDB confirming its role in mediating an anxiogenic phenotype. The frequency of anxiety disorders have been studied in monozygotic and dizygotic twins, in which the contribution of genetic factors ranges from 30 to 40%, while the rest can be attributed to differences in environment^3^. Thus, to study the gene–environmental interactions, we subjected HAB to EE and LAB were exposed to UCMS to shift their genetically predisposed anxiety phenotype towards normality.

HAB-EE mice were indeed found to have lower anxiety levels as indicated by higher percent time spent on open arm of EPM and light compartment of LDB in comparison to HAB-SH. While, LAB-UCMS mice had higher anxiety response compared to LAB-SH as indicated by lower percent time spent on open arm of EPM and light compartment of LDB. Intriguingly, in the aCC, we found that the HAB-EE had augmented *Tmem132d* mRNA expression compared to HAB-SH. On the other hand, LAB-UCMS had lower *Tmem132d* mRNA expression in comparison to LAB-SH. We also found significantly higher POLR2A occupancy at (−310)G locus of HAB-EE compared to HAB-SH, suggesting increased transcription, which explain the observed higher mRNA expression differences. On the other hand, there was significantly lower POLR2A binding at (−310)A locus of LAB-UCMS in comparison to LAB-SH, further explaining their attenuated level of gene expression. Thus, there is a bidirectional modulation of *Tmem132d* mRNA expression in HAB-EE and LAB-UCMS compared to their respective SH mice.

Studies have proposed that hippocampal neurogenesis^54^, synaptogenesis enhancement^55^, neuropeptideY^56^ and serotonin levels in frontal cortex^57^ may underlie EE induced decrease in anxiety. Thus, it is plausible that EE causes decrease in anxiety independent of *Tmem132d* in HAB mice. The exact regulatory function of *TMEM132D* gene is unknown.

**Fig. 6:**
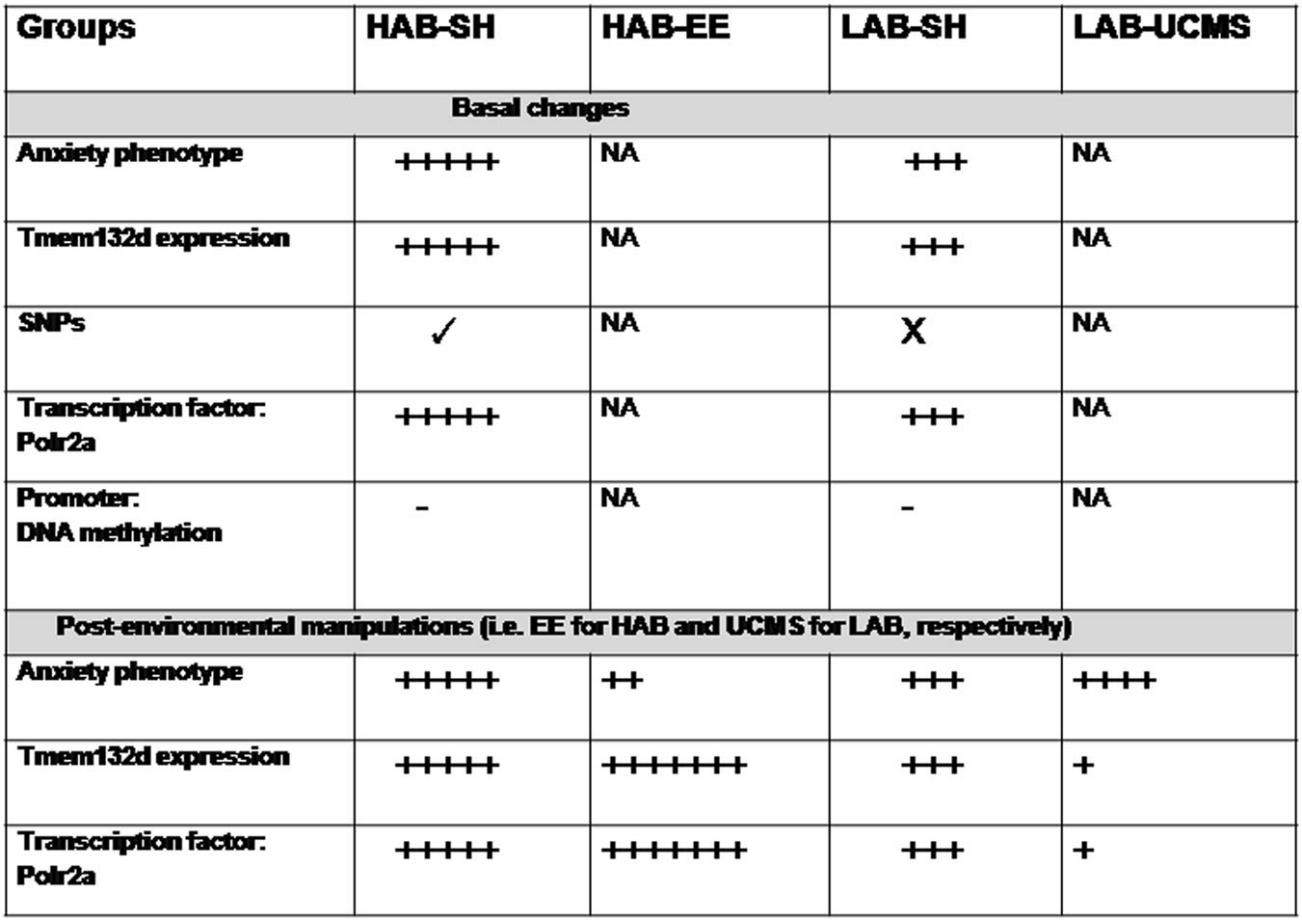
Summary of major behavioral and molecular events of HAB-SH/HAB-EE/LAB-SH/LAB-UCMS. Plus(+) sign(s) indicate increase in strength of a particular behavioral or molecular parameter. Minus (−) sign stands for no difference. NA stands for not applicable

From our previous studies, we predicted that an enhanced gene expression would lead to higher anxiety levels what holds true for the acute vector overexpression in mice. However, an early and prolonged positive environmental stimulation also seems to increase the gene expression levels in our animal model (HAB), whereas a prolonged mild stressful condition in LAB has opposite effects. This suggests time-dependent differential effects of the environment on *Tmem132d* gene regulation. Fig. 6 summarizes major behavioral and molecular alterations observed in our animal model.

In order to bring animal and human results together, we also looked for methylation difference in patients with PD in the *TMEM132D* gene region. The question was whether we can see any influence on methylation profile in dependence of life events in patients with risk alleles. We see two interesting associations, which show that patients homozygotic for the risk allele of rs7309727 show increased methylation in two CpGs with higher positive life events. In contrast, in patients having just one risk allele, we have an opposite effect. As increased methylation levels is mostly associated with lower gene expression. This is again in opposite to the findings in the HAB-EE mice. Further studies with analysis of control probands and different environmental exposure settings are needed to explain these effects. ENCODE project revealed POLR2A enrichment across *TMEM132D* gene body in K562 and GM78 cell lines, suggesting its importance in regulating *TMEM132D* gene expression^43,44^. RNA polymerase II pausing in proximal promoter region is widespread across genes involved in signal responsive pathways and has been described as a regulatory mechanism to diverse environmental cues in higher eukaryotes^58,59^. In summary, we see a modulatory function of environmental cues on *Tmem132d* gene expression in mice and methylation differences in risk allele patients with PD in response to positive life events, however, the direction of the changes remains unclear.

## Electronic supplementary material

supplementary section

Figure S2.1

Figure S2.2

Figure S2.3

Figure S3
