# Supplementary material for: Polymorphism in Tmem132d regulates expression and anxiety-related behavior through binding of RNA polymerase II complex
Source: Transl Psychiatry. 2018 Jan 10;8:1. doi: 10.1038/s41398-017-0025-2 (PMC5802467; doi:10.1038/s41398-017-0025-2)
Supplement: Supplementary file 1 — supplementary section [file 41398_2017_25_MOESM1_ESM.doc]

Naik et al

# Supplementary information:

**S1: Validation of putative transcription factor binding sites**

## Methods and Results: The plasmids for overexpressing nuclear factor I/C (NFI/C; clone ID: HsCD00041534) and general transcription factor IIB (GTFIIB; clone ID:HsCD00079856) were ordered from the Harvard PlasmID repository and cloned into pCSDest (Plasmid 22423, Addgene) using the Gateway® LR Clonase® II Enzyme mix (ThermoFisher Scientific). The shRNA constructs for NFI/C (TRCN0000310995) and GTFIIB (TRCN0000082049) were cloned into CaMKII-H1 promoter-empty-mcherry-AAV vector (kind gift from Prof. Li-Huei Tsai, Massachusetts Institute of Technology, USA), then transformed into suitable competent cells. Positive clones were amplified and purified plasmids were utilized for co-transfection experiments with HAB/LAB Tmem132d promoter constructs. Overexpression or knockdown of NFI/C and GTFIIB did not have any difference in HAB versus LAB Tmem132d promoter activity i.e. luciferase expression (data not shown).

**S2: Bisulfite sequencing of HAB/LAB Tmem132d promoter region**

**Methods and Results:** CpG islands are hotspots for DNA methylation and may be involved in differential regulation of Tmem132d, thus we utilized the CpG island searcher (http://cpgislands.usc.edu/) using the following settings: GC%: 55%, ObsCpG/ExpCpG: 0.65, length: 500bp and gap between adjacent islands: 100bp.

We discovered a 600bp CpG island in the putative Tmem132d promoter region and because it is a putative site for DNA methylation changes, we subjected genomic DNA from aCC of standard-housed HAB versus LAB to bisulfite conversion as follows: 400ng of genomic DNA was sheared 5x with a 26G needle (Josef Peske GmbH & Co. KG, Aindling, Germany) and subjected to bisulfite conversion using the EpiTect Bisulfite kit (Qiagen) as per manufacturer’s instructions. The primers (Table 8) for bisulfite sequencing were designed using default parameters at Methyl Primer Express v 1.0 (Life technologies, Darmstadt, Germany) software to cover the 600bp CpG island upstream from the transcription start site and then finally tailed with M13 forward and reverse primers to provide a universal primer binding site. Additional primers for genomic DNA tailed with M13 were made to verify for presence of any false positives due to incomplete bisulfite conversion.

PCR composition and conditions were adapted from Invitrogen handbook (cms_039258: http://tools.invitrogen.com/content/sfs/manuals/cms_039258.pdf).

Aliquots of bisulfite DNA were subjected to PCR with bisulfite specific or wild type primers. Bisulfite converted DNA covering 600bp of Tmem132d gene was amplified only with bisulfite primers (Figure S2.1).

There was no product observed with wild type primers suggesting complete bisulfite conversion.

Eventually, the products were purified and ligated into a pGEM®-T vector system (Promega GmbH, Mannheim, Germany) and transformed into chemically competent cells for blue/white screening. All the positive white colonies were prepared for cycle sequencing using a T7 universal primer on an ABI 3730 DNA analyzer (Life Technologies, Darmstadt, Germany). Finally, the bisulfite sequence reads were analyzed using BiQ Analyzer (Max Planck Institute for Informatics, Saarbrücken, Germany).

Overall, there was DNA methylation observed in the distal regions especially around the A(-519)G and A(-310)G SNP, which decreased towards the transcription start site (Figure S2.2).

Nonetheless, there was no difference in total percentage of methylation between standard-housed HAB versus LAB Tmem132d promoter (Figure S2.3), nor any difference at individual CpG positions between the two groups (data not shown).

Testing effects of DNA methylation on the Tmem132d promoter activity

# We cloned the Tmem132d promoter region into a CpG free luciferase vector (Kind gift from Prof. Michael Rehli, Dept. Internal Medicine III, University of Regensburg) and carried out dual luciferase (at least three independent) assays and could validate the results obtained earlier (data not shown). In addition, we carried out selective methylation (Table 9) of CpG dinucleotides adjacent to the SNPs using primers or complete methylation of the promoter region using CpG Methyltransferase (M.*SssI*) (New England Biolabs, Frankfurt am Main, Germany), however, this did not affect luciferase expression i.e. HAB/LAB Tmem132d promoter activity (data not shown).

# S3: Western blotting

To confirm the observed mRNA expression differences, aCC tissue from HAB-SH,LAB-SH along with samples from Tmem132d^(-/-)^ knockout (KO) mice were utilized to confirm antibody specificity (kind gift from Dr. Jan Deussing, MPIP, Munich). The total protein was extracted by homogenizing aCC tissue in RIPA buffer (Sigma) supplemented with Halt™ Protease and Phosphatase Inhibitor Cocktail (Thermofisher scientific) and incubated on ice for 30 min. Subsequently, the homogenate was centrifuged at 13000 rpm for 15 min at 4°C and the supernatant was collected and protein concentration estimation carried out using the Pierce BCA Protein Assay Kit (Thermofisher scientific). Equal amounts of protein (2-7µg) was loaded and resolved on a 10% SDS-PAGE, transferred onto a nitrocellulose membrane (Bio-Rad GmbH, Munich, Germany) and non-specific binding was blocked with 5% nonfat milk powder (Sigma-Aldrich) dissolved in Tris-buffered saline/0.1% Tween-20 (TBST), pH 7.4 for 1h at room temperature. Subsequently, the membranes were incubated with the following primary Tmem132d polyclonal antibodies: Bioorbyt-orb27852 (1:250), Novus biological LLC-NBP1-93563 (1:250), Abcam-ab116041 (1:1000) and Abgent-AP10011b (1:250).Except the Abcam antibody, all other antibodies were generated against the human TMEM132D but due to high degree of sequence homology with the corresponding murine protein, we utilized them. Subsequently, the blots were washed three times with TBST and incubated with respective HRP-conjugated secondary antibody (1:5000 or 1:10000 dilution) in 2.5% milk in TBST for 1h at room temperature. Finally, the blot was washed once again three times with TBST and visualized by chemiluminescence (Western Lighting, PerkinElmer).

All the four tested commercial antibodies showed similar bands between HAB, LAB and Tmem132d ^(-/-)^ KO (Figure S3). All the antibodies except one showed the expected band size for Tmem132d protein at 130kda. However, presence of an equally intense band at same position for Tmem132d ^(-/-)^ KO suggests non-specific nature of the tested antibodies.

The Bioorbyt (orb27852) antibody gave identical band patterns at 130, 100 and 70kda for HAB, LAB and Tmem132d^(-/-)^ KO sample (Figure S3.1). The Novus biological LLC (NBP1-93563) and Abcam (ab116041) antibodies also gave similar band pattern at 130 and 70kda size, respectively (Figure S3.2 and S3.3).The Abgent (AP10011b) antibody also gave similar band pattern at 130 and 100kda for HAB and KO sample while faint band pattern was observed for LAB sample (Figure S3.4). Thus, all the tested antibodies are non- specific and cannot be used to confirm observed mRNA expression differences.

**SI References:**

1. Takai D, Jones PA. Comprehensive analysis of CpG islands in human chromosomes 21 and 22. Proceedings of the National Academy of Sciences of the United States of America. 2002 Mar 19;99(6):3740-5. PubMed PMID: 11891299. Pubmed Central PMCID: 122594.

2. Takai D, Jones PA. The CpG island searcher: a new WWW resource. In Silico Biol. 2003;3(3):235-40. PubMed PMID: 12954087.
