# Supplementary figures and images for: Polymorphism in Tmem132d regulates expression and anxiety-related behavior through binding of RNA polymerase II complex
Source: Transl Psychiatry. 2018 Jan 10;8:1. doi: 10.1038/s41398-017-0025-2 (PMC5802467; doi:10.1038/s41398-017-0025-2)

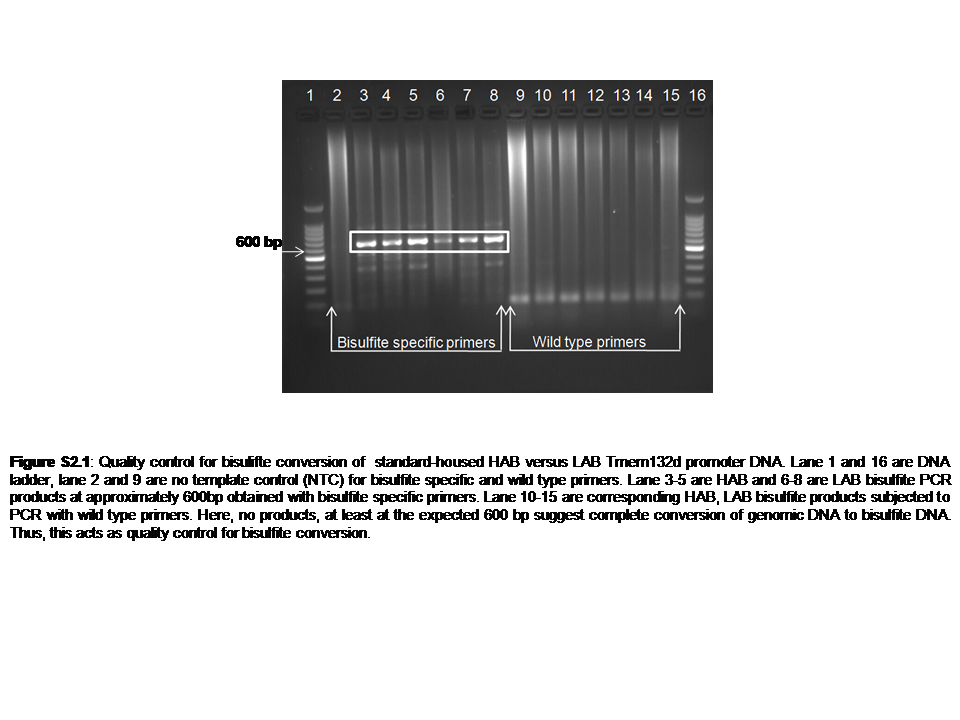

Supplement: Supplementary file 2 — Figure S2.1 [file 41398_2017_25_MOESM2_ESM.tif]

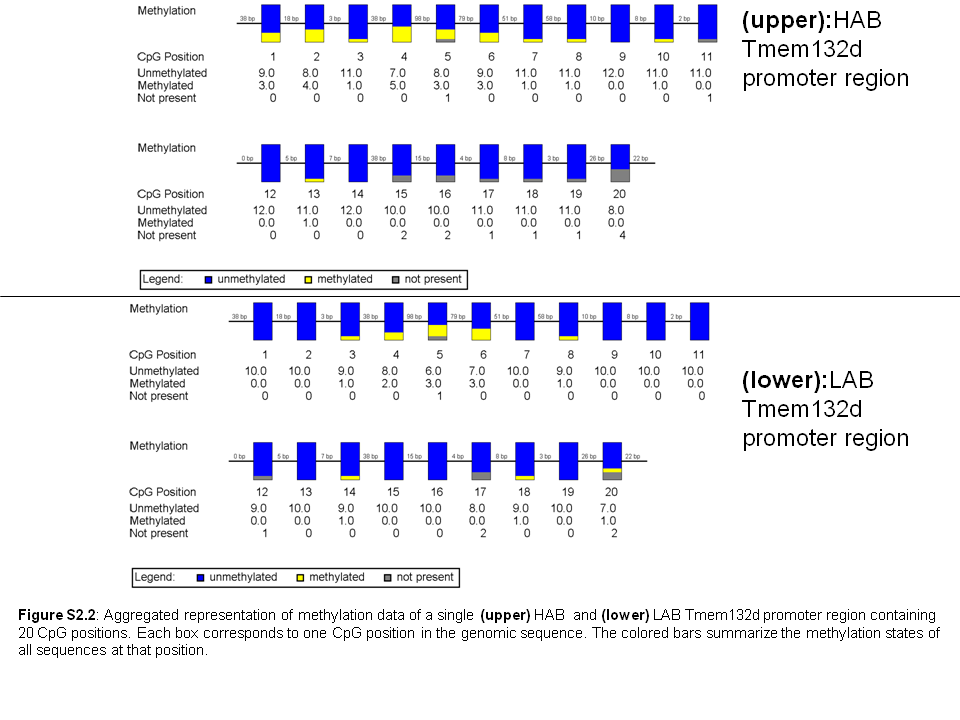

Supplement: Supplementary file 3 — Figure S2.2 [file 41398_2017_25_MOESM3_ESM.tif]

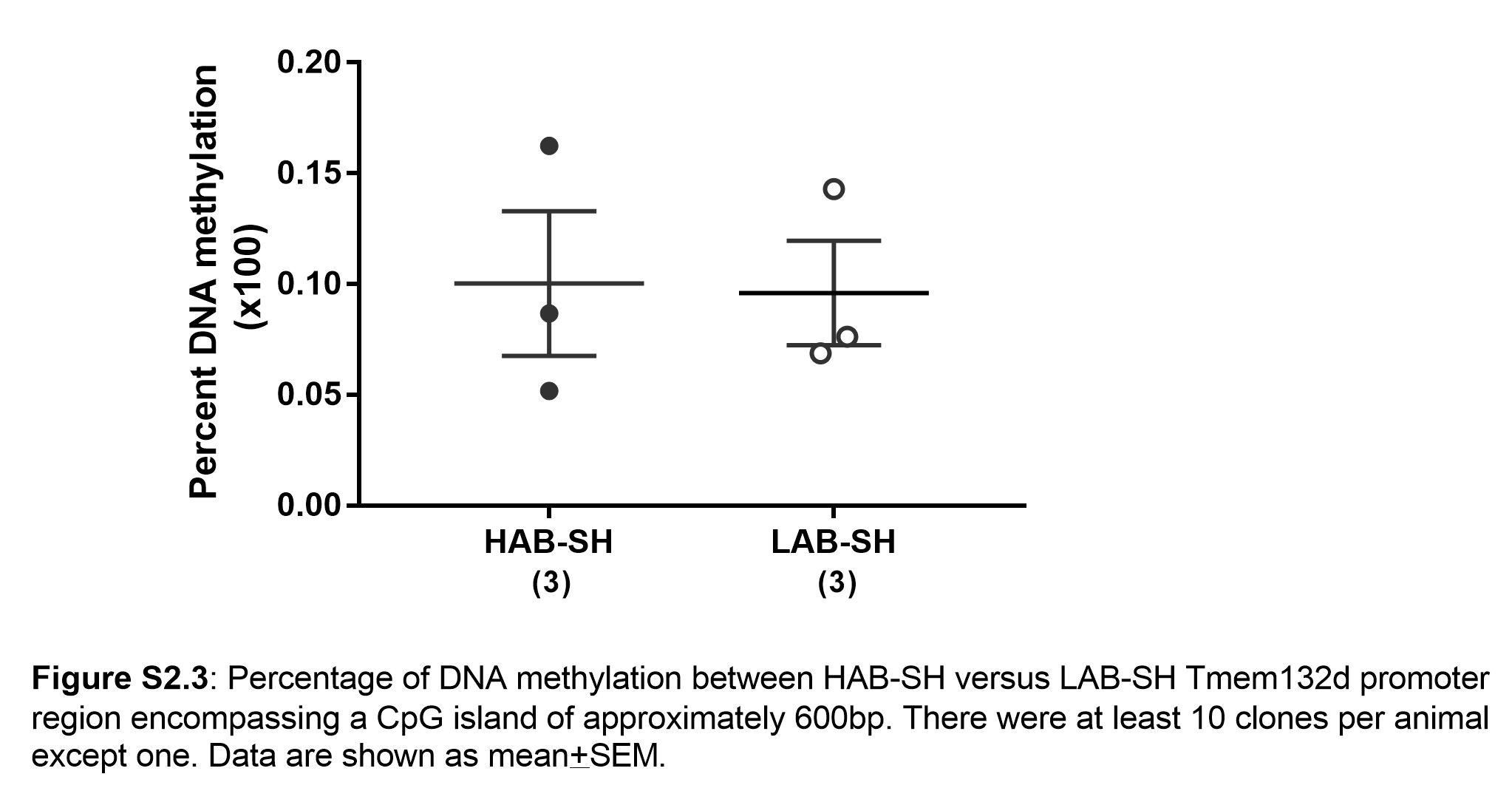

Supplement: Supplementary file 4 — Figure S2.3 [file 41398_2017_25_MOESM4_ESM.tif]

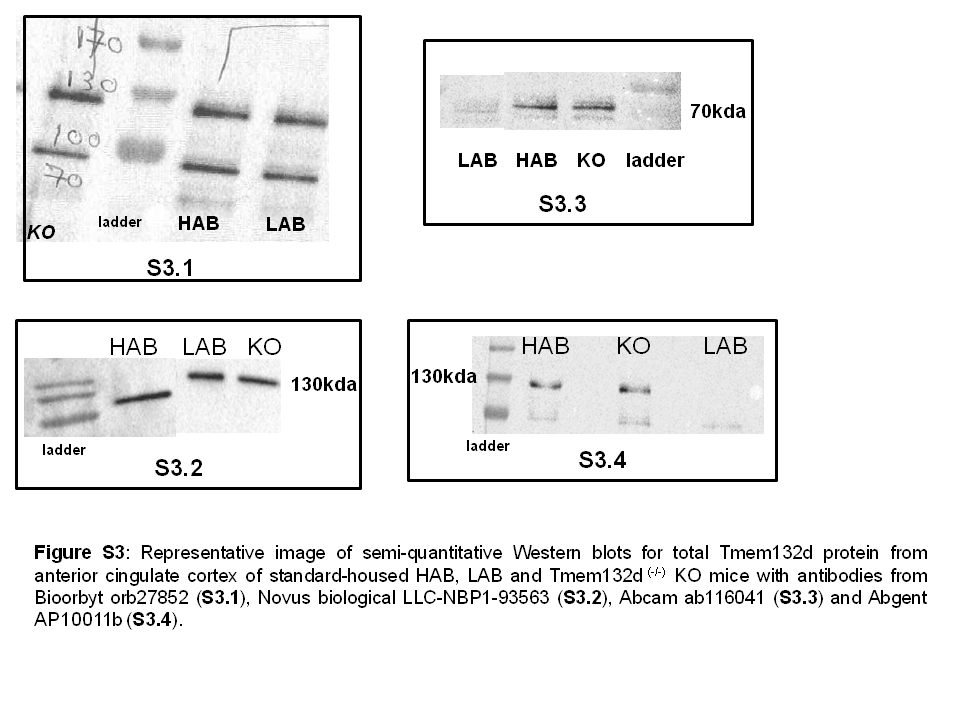

Supplement: Supplementary file 5 — Figure S3 [file 41398_2017_25_MOESM5_ESM.tif]
